# Immunometabolic Dysregulation in Preeclampsia: Emerging Roles of Inflammation, Insulin Resistance, Uric Acid, and the Gut Microbiome

**DOI:** 10.1155/mi/7890624

**Published:** 2026-07-16

**Authors:** Nurshad Ali

**Affiliations:** ^1^ Department of Biochemistry and Molecular Biology, Shahjalal University of Science and Technology, Sylhet, 3114, Bangladesh, sust.edu

**Keywords:** endothelial dysfunction, immunometabolism, inflammation, insulin resistance, preeclampsia, uric acid

## Abstract

Preeclampsia is a major cause of maternal and perinatal morbidity around the world. It is increasingly recognized as a disorder of systemic immunometabolic dysregulation rather than isolated placental dysfunction. Increasing evidence links chronic inflammation, insulin resistance, and changes in uric acid metabolism to the initiation and progression of preeclampsia. In addition, emerging evidence indicates that maternal gut dysbiosis is an upstream regulator of systemic immune and metabolic dysfunction via the gut–systemic–decidual axis. This review synthesizes current mechanistic, clinical, and translational evidence on the interplay between immune activation, metabolic dysfunction, and uric acid biology in relation to preeclampsia, highlighting emerging biomarkers and therapeutic implications. A narrative review was performed of experimental, epidemiological, and clinical studies found in peer‐reviewed journals. The review focused on pathways involving innate and adaptive immune activation, inflammation, insulin signaling abnormalities, endothelial dysfunction, and how uric acid affects placental and vascular biology. Preeclampsia shows increased activation of the innate immune system, a shift toward Th1/Th17 responses, vascular inflammation, and impaired immune tolerance. These immune disturbances combine with pregnancy‐associated insulin resistance, exacerbating oxidative stress and endothelial dysfunction, thereby reducing oxygen supply to the placenta. Elevated levels of serum uric acid (SUA), previously regarded as merely a marker of disease severity, are now thought to actively promote inflammasome activation, inhibit nitric oxide (NO), and disrupt trophoblast function. Together, these interconnected pathways form self‐reinforcing immunometabolic feedback loops that sustain vascular damage and drive the progression of the disease. Recent studies indicate that changes in the composition of maternal gut microbiota and their metabolites, such as short‐chain fatty acids (SCFAs) and endotoxins, can lead to systemic inflammation, endothelial dysfunction, and reduced immune tolerance. Immunometabolic dysregulation provides a comprehensive framework for understanding the pathogenesis of preeclampsia. Integrating inflammatory pathways, insulin resistance, serum uric acid, and alterations in the gut, systemic, and decidual microbiomes may improve risk stratification and facilitate the development of targeted preventive strategies. Nevertheless, well‐designed longitudinal and interventional studies are needed to validate these associations, establish causal relationships, and translate emerging evidence into effective prevention and management approaches across diverse populations.

## 1. Introduction

Preeclampsia is a hypertensive disorder that usually develops after 20 weeks of pregnancy, characterized by new‐onset hypertension, protein in the urine, or end‐organ dysfunction [1]. About 3%–8% of pregnancies worldwide are affected, and it is a major cause of morbidity and mortality for mothers and babies, especially in low‐ and middle‐income countries (LMICs) [2–4]. Despite decades of research, the pathophysiology of preeclampsia is still not fully understood, and delivery remains the only proven way to stop the disease from getting worse.

Preeclampsia has long been viewed as a placental disorder, caused by abnormal cell invasion and changes in blood vessels, leading to reduced oxygen delivery and the release of antiangiogenic and inflammatory factors into the material’s blood [5]. This view has helped researchers understand the disease, but it does not explain all the ways preeclampsia affects the body, its links to heart and metabolic dysfunction in mothers, or its long‐term effects [6]. Increasing evidence shows that preeclampsia is a multisystem disorder that affects especially immune regulation and metabolic homeostasis.

In a healthy pregnancy, the maternal immune system and metabolism adjust to support fetal growth and the mother’s health. Usually, the maternal body becomes more tolerant to the fetus, and mild insulin resistance facilitates nutrient transfer to the fetus [7]. In preeclampsia, these physiological changes are impaired. Studies demonstrated aberrant activation of the innate immune system, skewing of adaptive immune responses toward proinflammatory Th1 and Th17 phenotypes, and impaired regulatory T‐cell function [8, 9]. These immunological changes lead to systemic inflammation, endothelial activation, and oxidative stress, all key features of preeclampsia.

Concurrently, exaggerated insulin resistance and metabolic dysfunction are now seen as important features of preeclampsia. In most cases, women who develop preeclampsia already exhibit features of metabolic syndrome, such as obesity, abnormal cholesterol, and impaired glucose metabolism, even before pregnancy [10, 11]. At the molecular level, inflammation and oxidative stress disrupt insulin signaling, creating a bidirectional link between immune activation and metabolic abnormalities [12]. This immunometabolic interplay may increase vascular dysfunction and placental injury and accelerate disease progression.

Serum uric acid has long served as a marker for disease severity in preeclampsia. However, recent studies suggest that uric acid is not just a byproduct but plays an active role in the disease process. High levels of uric acid can cause endothelial dysfunction, reduce nitric oxide (NO) availability, activate the NLRP3 inflammasome, and hinder trophoblast invasion and blood vessel formation [13–15]. Uric acid is also closely associated with insulin resistance and chronic inflammation, positioning it as a potential molecular bridge between immune and metabolic pathways in preeclampsia.

The concept of immunometabolism now includes not only intracellular metabolic signaling but also interactions between the host and the microbiome. Studies in obstetrics support a “gut–systemic–decidual” model, where changes in the maternal gut microbiome can lead to systemic inflammation, metabolic dysfunction, and reduced immune tolerance [16, 17]. During pregnancy, shifts in gut microbes have been linked to higher intestinal permeability, movement of endotoxins like lipopolysaccharide (LPS) into the bloodstream, and lower levels of immunoregulatory metabolites such as short‐chain fatty acids (SCFAs) [18, 19]. These changes contribute to chronic low‐level inflammation, insulin resistance, and problems with blood vessel function, which are all important features of preeclampsia [20, 21] [20, 21].

In this expanded context, an immunometabolic framework provides a more comprehensive view of preeclampsia. It describes the condition as resulting from the overlap of inflammation, insulin resistance, and changes in uric acid metabolism. This review synthesizes existing mechanistic, clinical, and translational evidence supporting this paradigm, with the aim of identifying important knowledge gaps and discussing potential implications for assessing risk, prevention, and future therapeutic strategies.

A comprehensive literature search was performed in PubMed, Scopus, Web of Science, and Google Scholar for studies published up to 2025. The searching keywords were preeclampsia, inflammation, insulin resistance, uric acid, and immunometabolism. Where necessary, Boolean operators and MeSH terms were used. Original research articles, clinical studies, and pertinent reviews written in English were included. Studies that did not have mechanistic or clinical significance, nonpeer‐reviewed publications, and those with duplicated information were eliminated. The reference lists of the selected articles were also screened in order to find more relevant studies.

## 2. Preeclampsia as a Systemic Immunometabolic Disorder

Preeclampsia includes a range of clinical types that vary in timing, severity, and primary causes. It is usually divided into early‐onset preeclampsia (EOPE), which requires delivery before 34 weeks of pregnancy, and late‐onset preeclampsia (LOPE), which occurs at or after 34 weeks of pregnancy [22, 23]. EOPE is more often related to placental dysfunction, limited fetal growth, and negative outcomes during childbirth. In contrast, LOPE frequently links to the mother’s metabolic and cardiovascular factors. However, these categories are not separate, as both EOPE and LOPE can happen in women who have preexisting conditions like chronic hypertension, obesity, or metabolic dysfunction [22, 23]. This distinction highlights that preeclampsia is a varied syndrome arising from different yet context‐dependent factors.

A key aspect across this spectrum is the disruption of the maternal–placental–vascular axis, which connects placental signaling with maternal immune, metabolic, and endothelial responses. In normal pregnancy, adaptive vascular remodeling and metabolic adjustment support blood flow to the placenta and maintain healthy endothelial function. In preeclampsia, stress signals from the placenta—including factors that reduce blood vessel formation, inflammatory substances, and metabolic waste—interact with the maternal environment, which is affected by insulin resistance, abnormal fat levels, and immune activation, leading to widespread endothelial dysfunction [24, 25]. Notably, maternal vascular and metabolic abnormalities may appear before, worsen, or even cause placental pathology, particularly in LOPE.

Emerging evidence suggests that this axis should be extended to include the maternal gut microbiome. Gut‐derived inflammatory substances and metabolites can influence systemic immune activation, endothelial function, and placental signaling. Increases in circulating LPS and decreases in SCFAs, both linked to dysbiosis, have been associated with vascular inflammation and metabolic issues [17–19]. This suggests that the gut microbiota serves as an upstream regulator of the maternal–placental–vascular axis. This indicates a two‐way cycle of disease that is not solely due to the placenta.

Preeclampsia shares important features with chronic health issues, including inflammation in blood vessels, oxidative stress, problems with NO signaling, and metabolic rigidity [26]. These shared pathways support the well‐established link between preeclampsia and an increased long‐term risk of high blood pressure, heart disease, and type 2 diabetes in affected women [27]. From this angle, pregnancy serves as a sort of “stress test” revealing hidden immunometabolic vulnerabilities.

Understanding preeclampsia as a systemic immunometabolic disorder has important implications. It highlights the need to move beyond a solely placental‐centered view and consider the mother’s metabolic and inflammatory status when assessing the risk [5]. Clinically, preeclampsia is diagnosed based on new‐onset hypertension after 20 weeks of pregnancy along with proteinuria or signs of end‐organ dysfunction rather than directly examining placental health [1]. This perspective also supports developing preventive strategies that aim to improve immune and metabolic health before serious placental and vascular damage occurs [28, 29]. In this context, new immunometabolic biomarkers, like inflammatory mediators, insulin resistance measures, and uric acid‐related factors, may complement existing clinical criteria and help identify risks earlier and provide a clearer understanding of the disease [30, 31].

## 3. Immunological Dysregulation in Preeclampsia

Preeclampsia is now understood as a condition defined by significant immunological dysregulation that affects both innate and adaptive immunity (Table 1). Normal pregnancy requires a careful balance between the immune system accepting the fetus and protecting the mother from infections. In preeclampsia, this balance fails, leading to widespread inflammation, endothelial dysfunction, and increased oxidative stress [8, 9].

**Table 1:** Immunometabolic pathways implicated in the pathogenesis of preeclampsia.

Pathway/domain	Key molecular mediators	Mechanistic effects in preeclampsia	Primary target tissues	Evidence type	Strengths	Limitations	Key references
Innate immune activation	NF‐κB, NLRP3 inflammasome, IL‐1β, IL‐6, TNF‐α	Sustained systemic inflammation, endothelial activation, oxidative stress	Placenta, vascular endothelium	Human, experimental	Strong mechanistic evidence; consistent across animal and human studies	Low specificity; influenced by infection, obesity	[5, 15]
Gut microbiome dysbiosis	LPS, SCFAs, microbial metabolites	Systemic inflammation, insulin resistance, endothelial dysfunction	Gut–immune–vascular axis	Human + experimental	Strong emerging evidence	Heterogeneity, causality unclear	[18, 20, 21, 32]
Adaptive immune imbalance	Th1/Th17 cytokines (IFN‐γ, IL‐17), ↓ Treg cells	Loss of immune tolerance, vascular inflammation	Maternal immune system, placenta	Human	Explains maternal‐fetal immune interaction; validated in several cohort studies	Limited longitudinal data; heterogeneity across populations	[8, 9]
Insulin resistance	IRS‐1, PI3K/Akt, eNOS	Impaired NO production, vasoconstriction, hypertension	Vascular endothelium	Human, mechanistic	Mechanistic clarity; links metabolism to vascular dysfunction	Influenced by BMI, gestational age, ethnicity	[33]
Oxidative stress	ROS, NADPH oxidase, xanthine oxidase	Endothelial dysfunction, placental hypoxia	Placenta, endothelium	Experimental, clinical	Strong mechanistic evidence; widely replicated	Mostly associative in humans; difficult to measure clinically	[34]
Uric acid signaling	Uric acid, xanthine oxidase	Inflammasome activation, ↓ NO bioavailability, trophoblast inhibition	Placenta, kidney, endothelium	Human, experimental	Readily measurable; mechanistically central; integrates immune and metabolic pathways	Can be confounded by renal function; mostly correlative in early pregnancy	[13, 14]
Placental angiogenic imbalance	sFlt‐1, PlGF	Impaired placental vascular development, hypoperfusion	Placenta, maternal vasculature	Human	Strong clinical predictive value; validated biomarker assays	Limited early‐pregnancy prediction; cost and access issues	[29, 35]

Abbreviations: Akt, protein kinase B; eNOS, endothelial nitric oxide synthase; IFN‐γ, interferon gamma; IL, interleukin; IRS‐1, insulin receptor substrate‐1; NF‐κB, nuclear factor kappa B; NO, nitric oxide; NLRP3, NOD‐, LRR‐, and pyrin domain‐containing protein 3; PI3K, phosphatidylinositol 3‐kinase; ROS, reactive oxygen species; PlGF, placental growth factor; sFlt‐1, soluble fms‐like tyrosine kinase‐1; Th, T helper cell; TNF‐α, tumor necrosis factor alpha; Treg, regulatory T cell.

### 3.1. Innate Immune Activation

The innate immune system acts as the first line of defense during pregnancy. It responds through monocytes, neutrophils, natural killer (NK) cells, and dendritic cells. In preeclampsia, these immune cells become overactive. Monocytes and neutrophils show increased adhesion, higher production of reactive oxygen species, and more expression of inflammatory chemicals like tumor necrosis factor‐alpha (TNF‐α) and interleukin‐6 (IL‐6) [36]. NK cells, especially those in the uterus, are involved in the abnormal remodeling of spiral arteries. This dysfunction of NK cells leads to problems with placentation and local inflammation [37]. Additionally, toll‐like receptor (TLR) signaling is more active in women with preeclampsia, further raising the innate inflammatory response and promoting endothelial activation [38, 39].

### 3.2. Adaptive Immune Imbalance

In preeclampsia, the adaptive immune response changes because immune tolerance breaks down. This shifts from an anti‐inflammatory Th2/Treg state to a more proinflammatory Th1/Th17 profile. Regulatory T cells (Tregs) are important for maintaining maternal–fetal tolerance, but both their numbers and their function decline. As a result, there is more systemic inflammation and endothelial dysfunction [9, 40]. Recent studies indicate that Tregs and the complement system have a two‐way interaction that exacerbates immune dysregulation. Upon the activation of the complement system, complement products such as C3a and C5a have the potential to suppress Tregs and impair their capacity to regulate the immune response and also promote the development of Th17 cells [32, 41]. On the other hand, functional Tregs can help control excessive complement activation, indicating a feedback loop.

Excessive complement activation in preeclampsia can thus directly play a role in Treg dysfunction, compromising maternal immunological tolerance to fetal antigens [42]. This imbalance leads to chronic inflammation, vascular damage, and placental dysfunction. The interaction between complement signaling and adaptive immunity mechanistically links the innate immune response to tolerance loss in the pathology of immunology of preeclampsia [42, 43].

### 3.3. Inflammatory Mediators and Endothelial Dysfunction

In preeclampsia, elevated levels of proinflammatory mediators such as TNF‐α, IL‐6, IL‐1β, and IFN‐γ drive systemic inflammation. These mediators work together to damage endothelial function and increase both vascular permeability and blood clotting [24]. At the same time, overactivation of the complement system plays a key role in endothelial injury and immune imbalance. Increased levels of complement fragments, such as C3a, C5a, and the membrane attack complex (C5b‐9), are often found in preeclampsia and are associated with disease severity [42, 44].

Complement activation also affects adaptive immunity by altering the function of Treg cells. C5a signaling can weaken Treg cell suppression and increase inflammatory T‐cell activity, thereby sustaining endothelial inflammation [32]. When Treg regulation is disrupted, it leads to more cytokine production and further activates the endothelium. Damage to endothelial cells caused by complement also increases oxidative stress and lowers NO levels, which leads to blood vessel narrowing and high blood pressure [32, 45]. Overall, these findings show that the interaction between complement and Treg cells is a key factor in both immune imbalance and vascular dysfunction in preeclampsia.

### 3.4. Maternal–Placental Immune Crosstalk

The immune dysregulation in preeclampsia is closely related to stress in the placenta. When placental cells face oxidative or lack of oxygen stress, they release damage‐associated molecular patterns (DAMPs), which activate maternal innate immune receptors like TLRs and inflammasomes [25]. This activation boosts the systemic inflammatory response, leading to further endothelial dysfunction. Notably, immune problems in the mother may also occur before any visible placental damage appears. This suggests that the mother’s immune and metabolic vulnerabilities are both causes and results of the disease progressing [25].

### 3.5. Gut Microbiome and Immunometabolic Regulation

Recent advances have shown that the maternal gut microbiome is a key player in regulating immune and metabolic balance during pregnancy. The gut microbiota influences host immunity, metabolic signaling, and endothelial function through microbial metabolites and immune signals [16, 17].

In preeclampsia, several studies have found that microbial diversity decreases and the composition changes, leading to fewer beneficial microbes like lactobacillus and bifidobacterium and an increase in proinflammatory types. These changes are linked to greater intestinal permeability, which allows the movement of endotoxins like LPS into the bloodstream [18, 19]. Circulating LPS activates TLR4, boosting innate immune responses and triggering NF‐κB‐mediated inflammation. This process contributes to endothelial dysfunction and high blood pressure [46, 47]. At the same time, decreased production of SCFAs, especially butyrate, disrupts regulatory T‐cell differentiation and metabolic balance [48, 49].

These mechanisms driven by the microbiome are directly linked to insulin resistance, inflammasome activation, and vascular problems. They position the gut microbiota as an early initiator of the immune and metabolic changes seen in preeclampsia [20, 21].

## 4. Insulin Resistance and Metabolic Dysfunction in Preeclampsia

Metabolic adaptation is a key feature of normal pregnancy, ensuring proper nutrient delivery to the developing fetus. This adaptation includes a gradual increase in maternal insulin resistance during mid‐to‐late pregnancy, influenced by placental hormones and inflammatory signals [7]. In preeclampsia, these normal changes become exaggerated and disorganized. This leads to pathological insulin resistance and broader metabolic dysfunction, which contribute to endothelial injury, stress on the placenta, and the progression of the disease [50].

### 4.1. Exaggerated Insulin Resistance in Preeclampsia

Women who develop preeclampsia often show impaired insulin sensitivity well before clinical onset. Prospective studies demonstrated higher fasting insulin levels and increased homeostasis model assessment of insulin resistance (HOMA‐IR) during early pregnancy in women who later develop preeclampsia [51, 52]. This metabolic risk is especially noticeable in late‐onset diseases and aligns with common cardiometabolic risk factors like obesity, central adiposity, and dyslipidemia [53]. Importantly, insulin resistance in preeclampsia is not solely attributable to increased adiposity, indicating that there are internal flaws in metabolic signaling pathways.

### 4.2. Molecular Mechanisms Linking Insulin Resistance to Vascular Dysfunction

At the cellular level, insulin resistance in preeclampsia shows impaired signaling through the insulin receptor substrate (IRS)‐1/phosphatidylinositol 3‐kinase (PI3K)/Akt pathway. This pathway is crucial for activating endothelial NO synthase (eNOS) and producing NO. When insulin‐related NO signaling is disrupted, it leads to vasoconstriction, endothelial dysfunction, and high blood pressure, which are key features of preeclampsia [33]. Similarly, enhanced signaling through the mitogen‐activated protein kinase (MAPK) pathway increases the production of endothelin‐1 and blood vascular inflammation, further promoting constricted blood flow.

Inflammatory cytokines, such as TNF‐α and IL‐6, are significant in this process. They trigger the phosphorylation of IRS‐1, blocking insulin signaling [33, 54]. Oxidative stress, poor mitochondrial function, and excess free fatty acids worsen insulin resistance by hindering glucose uptake and lipid oxidation in maternal tissues [55]. Together, these changes create a metabolic environment that encourages endothelial injury and amplifies placental hypoxia.

The gut microbiota also plays an important role in regulating insulin sensitivity. Dysbiosis‐related endotoxemia leads to chronic low‐grade inflammation, which disrupts insulin signaling pathways, including IRS‐1/PI3K/Akt signaling [46, 56]. Decreased production of SCFA impairs glucose metabolism and enhances adipose tissue inflammation, which worsens insulin resistance in preeclampsia [48, 57].

### 4.3. Adipokines and Lipid Metabolism

Factors produced by the adipose tissue are increasingly recognized as important regulators of metabolic and vascular balance in pregnancy. In preeclampsia, circulating leptin levels are significantly elevated, reflecting both increased maternal adiposity and enhanced placental leptin production and secretion [58]. Leptin has proinflammatory and proangiogenic effects and is associated with trophoblast dysfunction and endothelial activation [59].

Alterations in lipid metabolism also define the metabolic profile of preeclampsia. Recent evidence from pregnant populations further indicates a high prevalence of dyslipidemia and related metabolic disturbances during pregnancy, reinforcing the role of lipid abnormalities in cardiometabolic risk [11]. High triglycerides, increased small dense low‐density lipoprotein particles, and increased lipid peroxidation lead to oxidative stress and endothelial dysfunction. Lipotoxicity within the placenta is linked to impaired mitochondrial function and an increase in inflammatory signals in maternal circulation, reinforcing systemic metabolic stress [60].

### 4.4. Placental Insulin Resistance and Metabolic Stress

The placenta also shows insulin resistance features in preeclampsia. Changes in glucose transporter expression, impaired insulin signaling, and mitochondrial dysfunction have been observed in placentas affected by preeclampsia, especially in early‐onset cases [61]. These issues hinder the placenta’s ability to manage nutrients and energy, worsening fetal growth restriction and oxidative stress. Additionally, placental metabolic dysfunction leads to the release of antiangiogenic factors and DAMPs, connecting metabolic stress to immune activation [61, 62].

### 4.5. Long‐Term Metabolic Consequences

The metabolic dysfunction seen in preeclampsia does not always resolve quickly after delivery. Women with a history of preeclampsia have a much higher risk of developing insulin resistance, type 2 diabetes, and metabolic syndrome later in life. Long‐term studies suggest that preeclampsia may speed up the onset of chronic metabolic diseases instead of just uncovering existing risks [63]. These findings further support viewing preeclampsia as a part of a continuum of cardiometabolic dysfunction throughout a woman’s life.

## 5. Uric Acid, From Biomarker to Pathogenic Mediator

Elevated uric acid, or hyperuricemia, has been known for a long time as a clinical indicator of preeclampsia severity and poor maternal‐fetal outcomes. Previously regarded as a result of decreased renal clearance or increased oxidative stress, serum uric acid is now seen as an active mediator that connects metabolic dysfunction, immune activation, and vascular injury. This change in understanding places uric acid at the center of the network that ties immune and metabolic issues together in preeclampsia.

### 5.1. Altered Uric Acid Metabolism in Preeclampsia

During a normal pregnancy, serum uric acid levels drop in early gestation due to increased renal clearance, followed by a gradual increase in late pregnancy. In preeclampsia, this normal pattern is interrupted, leading to early and persistent hyperuricemia [13]. Importantly, elevated uric acid often appears before clear clinical manifestations, suggesting that it plays a role in disease development rather than just reflecting disease pathogenesis.

Elevated uric acid in preeclampsia mainly arises from increased production and decreased kidney clearance. Placental low oxygen levels lead to more breakdown of ATP, which increases purine turnover and activates xanthine oxidase. This process contributes to more uric acid production and oxidative stress. At the same time, glomerular endotheliosis lowers the kidney filtration capacity, making it harder to excrete urate. Insulin resistance might further lower how well the kidneys clear uric acid. Emerging studies also suggest that changes in the gut microbiome may influence purine metabolism, potentially leading to high uric acid levels during pregnancy [13, 14, 18, 21]. These mechanisms together explain the higher production and lower elimination of uric acid seen in preeclampsia and support its role as both a marker and an active factor in disease development.

### 5.2. Uric Acid as a Driver of Endothelial Dysfunction

Studies show that uric acid directly impairs endothelial function. Soluble uric acid reduces the availability of endothelial NO by blocking eNOS activity and increasing oxidative stress. This process promotes vasoconstriction and hypertension [14]. In endothelial cells, uric acid increases the production of reactive oxygen species and boosts proinflammatory adhesion molecules, which helps white blood cells interact with the endothelium and causes vascular inflammation [14]. These effects align closely with the vascular phenotype of preeclampsia and are also observed in cardiometabolic disorders [14, 64].

### 5.3. Uric Acid and Immune Activation

Besides affecting blood vessels, uric acid also serves as a strong signal for the immune system. Soluble uric acid as well as monosodium urate crystals may activate innate immune pathways, in particular, the NLRP3 inflammasome. This process triggers caspase‐1 and increases the production of IL‐1β and IL‐18 [15, 65]. In preeclampsia, placental stress releases DAMPs, and uric acid further increases the innate immune activation and promotes systemic inflammation. This mechanism offers a molecular bridge connecting hypoxia in the placenta, immune dysregulation, and maternal endothelial damage [66, 67].

Uric acid also affects adaptive immune regulation and immune tolerance, which are important for a healthy pregnancy. An increased uric acid concentration has been reported to stimulate Th17 polarization and inhibit regulatory Treg differentiation and activity, thus altering the state of the immune system towards a proinflammatory phenotype [40, 68]. This imbalance directly disrupts maternal–fetal immune tolerance, which is vital for successful placental development. The suppressed Treg activity undermines fetal antigen tolerance, whereas augmented Th17 reactions upsurge IL‐17‐driven vascular inflammation and endothelial malfunction.

Furthermore, indirect amplification of adaptive responses through uric acid‐mediated inflammasome activation produces IL‐1 and IL‐18, which have been proven to stimulate Th1/Th17 responses and inhibit Treg stability [68, 69]. This defect in immune tolerance could explain a persistent maternal immune response to placental antigens, which further worsens trophoblast dysfunction and remodeling of spiral arteries.

The effect of uric acid in the activation of the inflammasome also overlaps with adaptive immunity, promoting Th17 polarization and inhibiting the activity of Tregs [67, 68]. This process enhances the proinflammatory immune environment of preeclampsia. These impacts add to immune alterations, positioning uric acid as an active contributor rather than a downstream bystander.

### 5.4. Interactions Between Uric Acid and Insulin Resistance

A growing body of evidence links hyperuricemia with insulin resistance and metabolic issues. Uric acid disrupts insulin signaling by causing oxidative stress, blocking the IRS‐1/PI3K/Akt pathway activity, and promoting endothelial dysfunction. These mechanisms relate to those described in metabolic syndrome and type 2 diabetes [70]. Conversely, insulin resistance decreases renal uric acid excretion, creating a self‐reinforcing loop between hyperuricemia and metabolic dysfunction [54, 71].

In pregnancy, this interaction is especially important. Insulin‐resistant states enhance xanthine oxidase activity and purine turnover, while uric acid exacerbates metabolic inflexibility and vascular dysfunction [72–75]. In preeclampsia, these combined effects intensify placental oxidative stress, endothelial injury, and immune activation, accelerating the disease progression.

Recent findings indicate that gut microbiota could affect uric acid metabolism by regulating purine metabolism and intestinal urate excretion. The connection between dysbiosis and elevated systemic levels of uric acid has been suggested to increase the systemic inflammasome response and oxidative stress in preeclampsia [76–78].

### 5.5. Effects on Placental Function

Uric acid has direct effects on placental biology as well. In vitro studies show that elevated uric acid levels hinder trophoblast invasion and disrupt angiogenic signaling, partly by reducing NO availability and increasing oxidative stress [79]. These effects hinder spiral artery remodeling and placental blood flow, reinforcing placental ischemia and continuing the release of inflammatory and antiangiogenic factors into the maternal bloodstream. Thus, uric acid contributes to a cycle connecting placental dysfunction with systemic immunometabolic stress.

### 5.6. Integrative Implications

**Figure 1:**
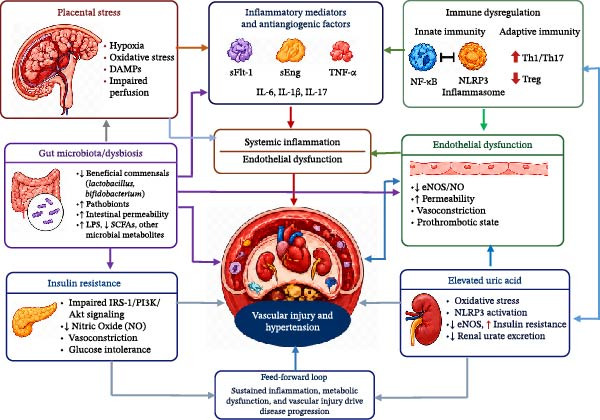
Immunometabolic crosstalk linking inflammation, insulin resistance, uric acid, and the gut–systemic–decidual axis in preeclampsia. This diagram illustrates interactions among placental stress, immune issues, insulin resistance, and uric acid. Gut microbiota imbalance acts upstream, promoting inflammation, metabolic problems, endothelial injury, and a self‐reinforcing cycle that leads to hypertension. Akt, protein kinase B; DAMPs, damage‐associated molecular patterns; eNOS, endothelial nitric oxide synthase; IL, interleukin; IRS‐1, insulin receptor substrate‐1; LPS, lipopolysaccharide; NF‐κB, nuclear factor kappa B; NLRP3, NOD‐, LRR‐, and pyrin domain‐containing protein 3; NO, nitric oxide; PI3K, phosphatidylinositol 3‐kinase; SCFAs, short‐chain fatty acids; sEng, soluble endoglin; sFlt‐1, soluble fms‐like tyrosine kinase‐1; Th, T helper cell; TNF‐α, tumor necrosis factor alpha; Treg, regulatory T cell.

Overall, these findings support an idea where uric acid acts as a key player in the immunometabolic aspects of preeclampsia. By promoting endothelial dysfunction, immune activation, and insulin resistance at the same time, uric acid ties together previously separate disease processes into a unified network. This role forms the basis for the immunometabolic interaction model shown in Figure 1, where inflammation, metabolic issues, and uric acid reinforce one another in a self‐sustaining cycle.

## 6. Immunometabolic Crosstalk: An Integrated Pathogenic Model

**Figure 2:**
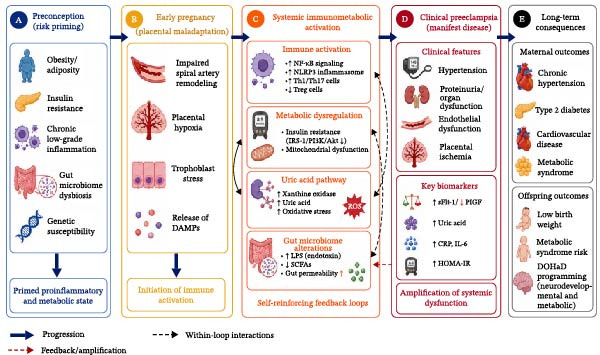
Stepwise progression of immunometabolic dysregulation in preeclampsia: from preconception risk factors to placental dysfunction, systemic activation, clinical disease, and long‐term maternal and offspring outcomes. Schematic showing the progression from preconception risk to placental stress and systemic immunometabolic activation, leading to clinical preeclampsia. It highlights feedback loops and the long‐term effects on maternal cardiovascular health and offspring metabolism. Abbreviations: NF‐κB, nuclear factor kappa B; NLRP3, NOD‐, LRR‐, and pyrin domain‐containing protein 3; Th, T helper cell; Treg, regulatory T cell; IRS‐1, insulin receptor substrate‐1; PI3K, phosphatidylinositol 3‐kinase; Akt, protein kinase B; LPS, lipopolysaccharide; SCFAs, short‐chain fatty acids; and PE, preeclampsia.

Growing evidence supports a single model in which preeclampsia develops from the active interaction between immune activation, metabolic dysfunction, uric acid metabolism, and signals from the microbiome, rather than from abnormalities within just one system [18, 28, 29]. This immunometabolic crosstalk model views preeclampsia as a self‐reinforcing network of pathological interactions involving the placenta, the maternal immune system, metabolic processes, and the vascular endothelium [9, 80]. A stepwise representation of this progression and its feedback loops are shown in Figure 2. Within this expanded framework, the maternal gut microbiome acts as a key regulator that can start or worsen immunometabolic problems. Dysbiosis‐driven endotoxemia and changes in microbial metabolites impact placental signaling, systemic inflammation, and blood vessel balance, integrating gut, systemic, and decidual axes into the development of preeclampsia [18, 19, 21].

At the center of this model is placental stress caused by poor blood flow, oxidative damage, and metabolic dysfunction. Stressed trophoblasts release inflammatory substances, antiangiogenic factors, and DAMPs into the maternal’s blood, starting and maintaining systemic immune activation [5]. These signals activate innate immune pathways, such as NF‐κB signaling and inflammasome assembly, while also shifting adaptive immunity toward more inflammatory Th1 and Th17 types [9]. The resulting mild systemic inflammation disrupts endothelial homeostasis and prepares maternal tissues for metabolic dysfunction.

Inflammation and metabolic stress are closely linked. Proinflammatory cytokines disrupt insulin receptor signaling by adding phosphate groups to IRS proteins, which causes increased insulin resistance. This insulin resistance further harms the function of blood vessels by hindering PI3K/Akt‐mediated NO production and triggering blood vessel narrowing [33]. These effects on blood vessels are key to the high blood pressure seen in preeclampsia and worsen placental blood flow, creating a cycle that adds to placental stress.

Uric acid plays a central role as a molecular amplifier in this network. High levels of uric acid reflect and worsen immunometabolic dysfunction. Hyperuricemia increases oxidative stress, activates the NLRP3 inflammasome, and reduces the bioavailability of endothelial NO, thus raising inflammation, insulin resistance, and blood vessel damage [14, 15]. In return, insulin resistance and inflammation reduce the kidney clearance of uric acid and increase xanthine oxidase activity, establishing a cycle that drives disease progression.

## 7. Clinical Implications

### 7.1. Biomarkers and Risk Stratification

Recognizing preeclampsia as a systemic immunometabolic disorder has important consequences for developing biomarkers and early risk assessment (Table 2). Inflammatory markers, such as C‐reactive protein (CRP), TNF‐α, IL‐6, and angiogenic imbalance markers, are consistently higher before and during the clinical disease. These markers reflect underlying immune activation and endothelial stress [5]. While each marker alone is not very specific, their predictive ability may improve when combined into multimarker panels. Current clinical and research biomarker strategies in preeclampsia mainly focus on angiogenic factors such as the sFlt‐1/PlGF ratio, which has significant diagnostic and short‐term predictive value, particularly in established cases of the disease [81, 85]. However, inflammatory markers and metabolic indices are not consistently included in routine clinical algorithms, and their independent predictive performance is limited when evaluated alone [5, 30].

**Table 2:** Immunometabolic biomarkers for risk stratification and prognosis in preeclampsia.

Biomarker category	Representative markers	Pathophysiological relevance	Clinical utility	Strengths	Limitations	Key references
Inflammatory markers	CRP, TNF‐α, IL‐6, IL‐17	Reflect systemic inflammation, immune activation, and endothelial injury	Risk stratification; disease severity and progression	Widely available assays; strong biological plausibility	Low specificity; influenced by infection, obesity, and gestational age	[9, 36]
Angiogenic imbalance	sFlt‐1, PlGF, sFlt‐1/PlGF ratio	Reflect placental stress and endothelial dysfunction	Diagnosis, short‐term prediction, clinical decision‐making	High predictive value in established disease; validated in trials	Limited early‐pregnancy prediction; cost and access constraints in LMICs	[35, 81]
Innate immune activation	NLRP3 inflammasome, IL‐1β, uric acid	Drives sterile inflammation and vascular injury	Mechanistic and emerging prognostic biomarkers	Integrates immune and metabolic pathways	Mostly research‐based; lack of standardized clinical assays	[67, 69]
Insulin resistance indices	Fasting insulin, HOMA‐IR, adiponectin	Reflect exaggerated pregnancy‐related insulin resistance	Early pregnancy risk assessment; cardiometabolic profiling	Measurable early; links maternal metabolic health to disease risk	Influenced by BMI, ethnicity, and gestational timing	[51, 52]
Metabolic dysregulation	Triglycerides, free fatty acids, leptin	Promote oxidative stress and endothelial dysfunction	Adjunctive metabolic risk stratification	Routinely measured; relevant to long‐term maternal risk	Poor specificity for preeclampsia alone	[82]
Uric acid‐related biomarkers	Serum uric acid (SUA), SUA/creatinine ratio (SUA/sCr)	Reflect oxidative stress, purine metabolism, endothelial dysfunction	Risk stratification and prognosis; SUA/sCr provides improved predictive accuracy	Widely available; SUA/sCr integrates renal function and metabolic stress	SUA alone confounded by renal function; ratio requires validation across populations	[13, 31, 83]
Endothelial dysfunction	NO metabolites, VCAM‐1, ICAM‐1	Reflect impaired vasodilation and vascular inflammation	Disease severity and progression	Directly reflects vascular pathology	Limited standardization; not routinely measured	[84]

Abbreviations: BMI, body mass index; CRP, C‐reactive protein; HOMA‐IR, homeostasis model assessment of insulin resistance; ICAM‐1, intercellular adhesion molecule‐1; IL, interleukin; IR, insulin resistance; NO, nitric oxide; NLRP3, NOD‐, LRR‐, and pyrin domain‐containing protein 3; PlGF, placental growth factor; ROS, reactive oxygen species; sFlt‐1, soluble fms‐like tyrosine kinase‐1; TNF‐α, tumor necrosis factor alpha; VCAM‐1, vascular cell adhesion molecule‐1.

Indices of insulin resistance, including fasting insulin, HOMA‐IR, adiponectin, and leptin, have shown predictive value in studies, especially for LOPE. These markers capture early metabolic vulnerability that often comes before noticeable hypertension and proteinuria. This supports their role in early‐pregnancy risk assessment among metabolically high‐risk populations [28, 86].

Serum uric acid (SUA) has long been used as a clinical marker in preeclampsia. However, its independent diagnostic and predictive value remain limited due to significant confounding by factors such as kidney function, gestational age, and maternal metabolic status [13, 30]. The most recent meta‐analyses and prospective cohort studies indicate that the serum uric acid‐to‐creatinine ratio (SUA/sCr) is a more accurate and physiologically relevant measure of true hyperuricemia [31, 87]. This ratio normalizes SUA to creatinine, helps adjust for pregnancy‐related changes in renal filtration, and better reflects oxidative stress, placental ischemia, and altered purine metabolism [31, 83, 88].

In addition, a large case–control study and meta‐analysis of prospective studies further support the limited specificity of SUA alone, strengthening the rationale for using normalized indices such as SUA/sCr in risk stratification [87]. Importantly, the SUA/sCr ratio has been shown to be more predictive of adverse maternal and fetal outcomes than SUA alone, especially in the initial disease stratification and severity [31, 83, 88]. By integrating renal function, metabolic stress, and vascular injury, this ratio may serve as an effective biomarker for predicting risk in heterogeneous populations. Future clinical guidelines should focus on composite or ratio biomarkers, such as SUA/sCr, rather than relying solely on SUA levels.

Conversely, the immunometabolic framework suggested in this review integrates these domains by combining inflammatory mediators, indices of insulin resistance, uric acid‐linked measures, and novel microbiome‐linked metabolites [9, 16, 17]. Such multimarker methods may offer an earlier identification of disease vulnerability and be more reflective of the upstream pathophysiological mechanisms that lead to overt clinical manifestations [18, 21, 28].

### 7.2. Therapeutic Perspectives

Current clinical management of preeclampsia mainly focuses on controlling symptoms and preventing complications. This includes using antihypertensive therapy, administering magnesium sulfate to prevent seizures, and ensuring timely delivery, which is the only definitive treatment [1].

Understanding the interactions between immunology and metabolism opens up new pathways for prevention and treatment. However, putting these ideas into clinical practice requires careful thought about timing and safety. Anti‐inflammatory strategies are getting more attention. Low‐dose aspirin is currently the most commonly recommended preventive measure for women at high risk of preeclampsia. Large randomized trials and meta‐analyses have shown that starting low‐dose aspirin, especially before 16 weeks of pregnancy, greatly lowers the chances of developing preeclampsia, particularly the preterm form [89–91]. Its beneficial effects likely involve changes in placental blood flow, inflammation, and endothelial function [90, 91]. Based on this evidence, major clinical guidelines now suggest low‐dose aspirin for high‐risk groups.

Strategies that enhance insulin sensitivity, such as lifestyle changes targeting weight, diet, and exercise before or early in pregnancy, are low‐risk options with potential benefits. Medications that improve insulin sensitivity, like metformin, show promise in reducing metabolic stress and endothelial dysfunction. However, the evidence for preventing preeclampsia is mixed, and routine use is not currently recommended outside specific indications [92].

Interventions to lower uric acid could be a promising target due to its role as an immunometabolic amplifier. Experimental studies suggest that blocking xanthine oxidase might lower oxidative stress and improve endothelial function. However, clinical data on the safety and effectiveness of uric acid‐lowering treatments during pregnancy are limited. Concerns about fetal safety currently prevent routine use [14].

Timing and safety are very important. Interventions are more likely to work if applied early before irreversible damage occurs to the placenta and blood vessels. Therefore, identifying risks before conception and early in pregnancy, along with low‐risk preventive strategies, may have the greatest clinical impact. Future trials should focus on early intervention windows, multimodal risk assessment, and long‐term maternal cardiovascular outcomes.

## 8. Long‐Term Maternal and Offspring Health Consequences

Preeclampsia is increasingly seen as a key event that has lasting effects on both maternal and offspring health. Women with a history of preeclampsia face a significantly higher risk of developing chronic hypertension, ischemic heart disease, stroke, type 2 diabetes, and metabolic syndrome later in life. Meta‐analyses show a two‐ to fourfold increase in cardiovascular disease risk, lasting for decades after the affected pregnancy [63, 93]. These links cannot be fully explained by shared risk factors, indicating that preeclampsia itself contributes to long‐term vascular and metabolic damage.

The immune and metabolic issues that come with preeclampsia—chronic inflammation, insulin resistance, problems with blood vessel function, and high uric acid levels—reflect key processes behind cardiometabolic diseases. Ongoing changes in blood vessel response, metabolic control, and immune signaling after delivery support the idea that preeclampsia speeds up a negative cardiometabolic path rather than just uncovering preexisting risk [27]. This shared biology connects complications during pregnancy to chronic diseases later in life.

The negative effects are not limited to the mother. Offspring from preeclamptic pregnancies have higher risks of low birth weight, preterm birth, and poor fetal growth. These factors are linked to a greater likelihood of hypertension, insulin resistance, and cardiovascular disease in adulthood. This pattern supports the developmental origins of health and disease (DOHaD) hypothesis, which states that exposure to inflammation, oxidative stress, and metabolic issues in the womb increases long‐term disease risk [94]. Growing evidence shows that stress in the placenta might lead to changes at the epigenetic level that affect vascular, metabolic, and immune functions in the offspring.

## 9. Global and Health‐System Perspectives

Preeclampsia remains a leading cause of maternal and perinatal complications and deaths worldwide, and LMICs bear a disproportionate burden. Limited access to early antenatal care, delayed diagnosis, and weak referral systems lead to preventable complications and deaths. LMICs are also undergoing a rapid epidemiological shift, which includes rising obesity, insulin resistance, and noncommunicable diseases, alongside undernutrition and micronutrient deficiencies. This situation creates a fertile ground for immunometabolic risk clustering, increasing the likelihood of preeclampsia [3].

Nutritional inadequacies, chronic infections, environmental stress, and metabolic issues often come together in LMIC populations. Diets rich in refined carbohydrates and poor in micronutrients, combined with a lack of physical activity and socioeconomic stress, foster systemic inflammation and insulin resistance even before pregnancy. These elements intersect with pregnancy‐related immunometabolic changes, raising the risk of hypertensive disorders. High inflammatory markers, poor glucose metabolism, and elevated uric acid levels can often be detected early in pregnancy, providing chances for low‐cost risk assessment using common tests [95].

From a health‐system viewpoint, understanding preeclampsia as a systemic immunometabolic disorder encourages a focus on early screening and prevention rather than just late‐stage management. Adding simple biomarkers—like blood pressure, body mass index, fasting glucose, and serum uric acid—to antenatal care packages could improve early risk identification in resource‐limited areas. Preventive strategies that stress preconception care, nutritional improvement, weight control, and low‐dose aspirin for high‐risk women are especially pertinent for LMICs, where advanced obstetric options may not be available [91].

Enhancing antenatal monitoring through community programs and shifting tasks to primary care providers can lead to better early detection and referral. Addressing preeclampsia within broader maternal cardiometabolic health frameworks fits with global efforts to reduce maternal mortality and control the growing burden of noncommunicable diseases throughout life.

## 10. Knowledge Gaps and Future Research Directions

Despite significant progress in understanding the immunometabolic features of preeclampsia, important knowledge gaps remain that hinder the development of effective prevention and treatment strategies. A key unresolved question is the difference between causality and association. While inflammation, insulin resistance, and high uric acid levels are consistently seen in preeclampsia, it is still unclear if these issues trigger the disease or mainly reflect the consequences of placental dysfunction. To clarify cause‐and‐effect relationships, we need study designs that can capture the timing and interactions of different mechanisms rather than just snapshot data [5].

The timing of immunometabolic dysregulation is another significant gap. Evidence indicates that metabolic and inflammatory disruptions may occur in early pregnancy or even before conception, especially in women who later develop LOPE. However, the exact time frame during pregnancy when immune, metabolic, and uric acid pathways start to differ from normal patterns remains poorly understood. Finding strong early‐pregnancy biomarkers, ideally easy and inexpensive to measure in the first trimester, is crucial for effective risk assessment and preventive measures [34].

The role of the maternal gut microbiome in preeclampsia is not fully understood. Important gaps include the cause‐and‐effect relationship, changes over time during pregnancy, and the potential benefits of interventions that target the microbiome, such as probiotics, prebiotics, and dietary modifications.

There is also an urgent need for long‐term human studies that combine clinical profiling with molecular, immune, and metabolic assessments throughout pregnancy and into the postpartum period. Most current data come from late‐pregnancy samples or experimental models that do not fully capture human diseases. Prospective studies that analyze biomarkers over time along with placental and vascular functions would provide valuable insights into how the disease evolves and varies [29].

Lastly, the potential for targeted immunometabolic treatments is still largely unexamined. While low‐dose aspirin and lifestyle changes offer some benefits, future strategies may need to focus on specific pathways, such as those driven by inflammation versus those driven by metabolism. Before we can safely implement these treatments in pregnancy, we must rigorously assess the safety for both mother and baby, determine the best timing, and evaluate their long‐term effects. Progress in this area will depend on collaboration across multiple disciplines, including obstetrics, immunology, metabolism, and cardiovascular research.

## 11. Conclusion

Preeclampsia is increasingly seen as a systemic immunometabolic disorder instead of just a placental issue. The evidence presented in this review shows how immune dysfunction, insulin resistance, changes in uric acid metabolism, and the gut microbiome are key, interconnected factors that drive endothelial dysfunction, placental insufficiency, and high blood pressure. Stress on the placenta triggers inflammatory and metabolic signals that interact with the mother’s susceptibility. Uric acid acts as an important molecular connector that links immune activation to metabolic and vascular damage. These interconnected pathways create self‐reinforcing loops that explain the variability in the disease, its progression, and its long‐term effects on heart health and metabolism.

This broader view of immunometabolism, including the role of the microbiome, indicates important clinical and practical implications. It supports earlier risk assessment using combined biomarker profiles, guides preventive measures targeting metabolic and inflammatory pathways, and offers a framework to understand the increased cardiovascular risk seen after preeclampsia. This model highlights the potential benefits of interventions applied before permanent damage occurs to the placenta and blood vessels, especially in high‐risk groups.

Advancing this field will require coordinated multidisciplinary research integrating longitudinal human studies, mechanistic investigations, and systems biology approaches. Collaboration across obstetrics, immunology, metabolism, cardiovascular medicine, and global health is essential to translate immunometabolic insights into effective strategies for the prevention, early detection, and management of preeclampsia. Adopting a life‐course and systems biology perspective offers a promising opportunity to improve maternal and offspring health and reduce the long‐term burden of preeclampsia.

## Author Contributions

**Nurshad Ali**: conceptualization, writing – original draft, writing – review and editing.

## Funding

The author received no specific funding for this work.

## Conflicts of Interest

The author declares no conflicts of interest.

## Data Availability

No new data was generated for the research described in the article.
